# Immunomodulatory function and anti-tumor mechanism of natural polysaccharides: A review

**DOI:** 10.3389/fimmu.2023.1147641

**Published:** 2023-03-09

**Authors:** Yang Ying, Wu Hao

**Affiliations:** ^1^ Cancer Institute, The Second Affiliated Hospital, Zhejiang University School of Medicine, Hangzhou, Zhejiang, China; ^2^ Key Laboratory of Cancer Prevention and Intervention, China National Ministry of Education, Key Laboratory of Molecular Biology in Medical Sciences, Hangzhou, Zhejiang, China; ^3^ Zhejiang Provincial Clinical Research Center for Cancer, Cancer Center of Zhejiang University, Hangzhou, Zhejiang, China

**Keywords:** polysaccharides, anti-tumor, tumor, immunomodulatory, immune response

## Abstract

Polysaccharides extracted from natural resources have attracted extensive attention in biomedical research and pharmaceutical fields, due to their medical values in anti-tumor, immunomodulation, drug delivery, and many other aspects. At present, a variety of natural polysaccharides have been developed as adjuvant drugs in clinical application. Benefit from their structural variability, polysaccharides have great potential in regulating cellular signals. Some polysaccharides exert direct anti-tumor effects by inducing cell cycle arrest and apoptosis, while the majority of polysaccharides can regulate the host immune system and indirectly inhibit tumors by activating either non-specific or specific immune responses. As the essential of microenvironment in the process of tumor development has been gradually revealed, some polysaccharides were found to inhibit the proliferation and metastasis of tumor cells *via* tumoral niche modulation. Here, we focused on natural polysaccharides with biomedical application potential, reviewed the recent advancement in their immunomodulation function and highlighted the importance of their signaling transduction feature for the antitumor drug development.

## 1 Introduction

It has been estimated that there were 19.3 million new cancer cases and almost 10 million cancer deaths worldwide per year (1). People’s physical and mental health is being threatened by its high incidence and high mortality and patients suffered from and mostly died of progressive failure of multi organ systems. Current, treatment methods or include surgery, radiotherapy, chemotherapy and immunotherapy. Although these methods have certain therapeutic advantages on early tumors, they are often ineffective in patients with advanced and metastatic tumors, and in many cases, they have serious side effects (2). This prompts researchers to look for new anti-tumor drugs/methods with lower toxicity, higher efficiency and fewer side effects (3).

Bioactive components extracted from natural resources such as macrofungi, plants, animals and microorganisms have been proven to have great potential in the prevention and treatment of cancer (4–6). These active components mainly contain polysaccharides, glycopeptides/protein complexes, proteoglycans, proteins, triterpenes and so on (7). Polysaccharides are a class of natural polymer formed by connecting aldose or ketose with glycoside bond (8). Compared with the amino acids in proteins which are only interconnected in one way, the monosaccharide units in polysaccharides are able to be interconnected at several points to form a wide range of branched or linear structures. This structural diversity is almost unlimited, which gives the necessary flexibility to the precise regulatory mechanisms of various cell-cell interactions in higher organisms (9). In addition, a large number of studies have indicated that these natural polysaccharides have significant anti-tumor effects without obvious side effects (2, 10). Therefore, a diversity of natural polysaccharides, such as *Astragalus* polysaccharide, *Ginseng* polysaccharide, lentinan, fucoidan, *Coriolus versicolor* polysaccharide and pachman, have already become clinical drugs (3, 11, 12).

This review focused on natural polysaccharides with biomedical application potential, reviewed the recent advancement in their immunomodulation function and highlighted the importance of their signaling transduction feature for the antitumor drug development.

## 2 Structure features

Polysaccharides are constructed by a large number of monosaccharides linked through glyosidic bonds. The monosaccharide units mainly consist of glucose, galactose, mannose, xylose, arabinose, caramel, ribose and glucuronic acid (13). Polysaccharides extracted from natural resources are mostly heterogeneous, i.e., heteropolysaccharides composed of different monosaccharides, except for a few homopolysaccharides. Most polysaccharides with strong biological activity have αβ-helix structure (10). Among them, β-D-glucan are deemed to be the most important and potent immunomodulating polysaccharides, and several linear and branched β-D-glucan have been reported to have great biological activity latent capacity (14). The glycosidic bonds of plant-derived polysaccharides are primarily α-(1→6)-D, α-(1→4)-D and β-(1→4)-D. However, even the polysaccharides separated from the same plant may be of vast difference (10). For example, two fractions F1 and F2 can be extracted from *Schizophyllum commune*. The F1 fraction was composed of glucose (75.5% and 88.2%) with small amounts of mannose, galactose and xylose, while the F2 fraction was comprised of mannose (55.2%) with minor amounts of galactose, glucose, and xylose. Moreover, F2 has stronger immunomodulatory activity (15).

As almost all physiological activities rely on the aqueous environment, the solubility of polysaccharides in water affects their biological function to a considerable extent. The water solubility of polysaccharides depends on many factors. Structures that hinder the intermolecular association usually lead to a higher solubility, such as branching structures and charged groups (carboxylic acid group, sulfate group or phosphate group) (16–18); on the contrary, the structural features that promote intermolecular association result in poor solubility, such as linear chain, large molecular weight, and other regular structural characters (19). This provides an idea for how to take full advantage of the biological activity of natural polysaccharides. Thus, many polysaccharides products with chemical modifications (e.g. carboxymethylation, hydroxylation, formyl methylation, amination and sulfation) have been designed and applied to the market (20).

**Table 1** lists the structural features and possible anti-tumor mechanisms of some natural polysaccharides purified from fungi, plants, animals and microorganisms. Interestingly, most of the natural polysaccharides that can directly act on tumor cells rather than play an anti-tumor role through immune regulation are highly water-soluble polysaccharides with charged groups. For example, both *Bupleurum chinense* polysaccharide BCP (43) and *Salvia miltiorrhiza* polysaccharide SMP (52) are acidic polysaccharides, and both of them could cause cell cycle arrest and induce apoptosis. Ginger polysaccharide GP was found to have a triple helical structure (57). Some literatures had reported that polysaccharides with triple helix structure have high antitumor activity (57, 65), but the exact relationship between the triple helix structure and antitumor function of polysaccharides is still unclear (57). However, this can still shed some light on us and provide experience for subsequent analysis of the association of polysaccharides structure and biological activity.

**Table 1:** Structural features and anti-tumor mechanism of some polysaccharides from nature resources.

Type	Species name	Polysaccharide(s)	Structural feature (s)	Anti-tumor mechanism	Reference (s)
Macrofungus	*Craterellus cornucopioides*	CCP	(1→3)-β-D-Manp- (1→6)-α-D-Galp	Immunomodulation: Macrophage	(21)
	*Coriolus versicolor*	PSK	Protein-bound polysaccharide	Cell-cycle arrest and apoptosis induction Immunomodulation: NK cell	(22)
	*Dictyophora indusiata*	DP1	(1→3)-α-L-Man, (1→2,6)-α-D-Glc	Immunomodulation: Macrophage	(23)
	*Entoloma lividoalbum*	ELPS	(1→3,6)-β-D-Glcp, (1→3)-β-D-Glcp, (1→6)-β-D-Glcp	Anti-oxidation Immunomodulation: Macrophage	(24)
	*Flammulina velutipes*	FVP1	Homogeneous polysaccharide	Immunomodulation: Macrophage	(25)
	*Ganoderma atrum*	PSG-1	Protein-bound polysaccharide	Anti-oxidation Immunomodulation: Macrophage and T cell	(26, 27) (28–31)
	*Ganoderma lucidum*	GLIS	Proteoglycan	Immunomodulation: B cell	(32)
	*Lentinus fusipes*	PS-II	(1→6)-α-D-galactan, (1→6)-β-D-glucan	Anti-oxidation Immunomodulation: Macrophage	(4)
	*Phellinus baumii*	PPB	Homogeneous polysaccharide	Cell-cycle G0/G1 arrest	(33)
	*Phellinus ribis*	PRP-S1, PRP-S2	(1→4)-, (1→6)-β-glucan *sulfated derivatives	Anti-angiogenic effect	(34)
	*Pleurotus citrinopileatus*	PCP	Pyranose (α-glucan & β-glucan)	Cell-cycle S arrest and apoptosis induction	(35)
	*Pleurotus ostreatus*	Se-POP-3	Selenium-enriched heteroglycan	Apoptosis induction	(36)
	*Polyporus umbellatus*	PPS	D-glucan	Immunomodulation: DCs, T cell and NK cell Adjuvant chemotherapeutic drugs	(37) (38, 39)
	*Schizophyllum commune*	F2	(1→3)-mannan, (1→2,3)-galactan	Immunomodulation: Macrophage	(15)
	*Tricholoma lobayense*	TLH-3	(1→3)-α-D-glucan	Anti-oxidation	(40)
Plant	*Alfalfa*	APS	Heteroglycan	Immunomodulation: B cell	(41)
	*Artemisia sphaerocephala*	ASPs	Acidic heteroglycan	Cell-cycle S arrest and apoptosis induction	(42)
Plant	*Bupleurum chinense* DC	BCP	Acidic heteroglycan	Cell-cycle S arrest and apoptosis induction	(43)
	*Codium fragile*	CFP	Sulfated polysaccharide	Immunomodulation: DCs, NK cell, T cell	(44, 45)
	*Codonopsis pilosula*	CPPS	Heteroglycan	Immunomodulation: T cell	(46)
	*Gayralia brasiliensis*	Gb1 Gb1-OS	Sulfated polysaccharide Over-sulfated polysaccharide	Cell-cycle G1 arrest Cell-cycle S and G2 arrest	(47)
	*Hippophae rhamnoides*	HRWP-A	(1→4)-β-D-galactan	Immunomodulation: Macrophage Adjuvant chemotherapeutic drugs	(48)
	*Laminaria japonica*	LJP-31	Homogeneous polysaccharide	Immunomodulation: Macrophage	(49)
	*Nemalion helminthoides*	N3, N4	(1→3)-α-D-mannopyranose	Immunomodulation: Macrophage	(50)
	*Ophiopogon japonicus*	OPL	Polysaccharide liposome	Immunomodulation: Macrophage	(51)
	*Salvia miltiorrhiza*	SMP	Acidic heteroglycan	Cell-cycle S arrest and apoptosis induction Anti-oxidation	(52)
	*Sargassum fusiforme*	SFPS	Heteroglycan	Immunomodulation: Macrophage Anti-angiogenic effect Apoptosis induction	(53) (54) (5)
	*Tarphochlamys affinis*	PTA	Heteroglycan	Immunomodulation: T cell, NK cell Apoptosis induction	(55)
	*Tinospora cordifolia*	G1-4A	Heteroglycan	Immunomodulation: Macrophage	(56)
	*Zingiber officinale* (Ginger)	GP	Heteroglycan with a triple helix	Cell-cycle G0/G1 arrest and apoptosis induction	(57)
Animal	*Edwardsia sipunculoides*	SAP30/60/80	Heteroglycan	Anti-oxidation	(6)
	*Philomycus bilineatus*	PBP60-C PBP60-D	Heteroglycan	Anti-oxidation	(58)
	*Scolopendra subspinipes mutilans L. Koch*	SPPC	polysaccharide–protein complex	Immunomodulation	(59)
Microorganism	*Alternaria mali* Roberts	AMEP-2	Manp-(1→4) and Glcp-(1→6)	Cell-cycle arrest and apoptosis induction	(60)
	*Hirsutella sinensis*	HSP-III	(1→3)glucose	Apoptosis induction	(61)
	*Morchella esculenta*	MP-1/3/4	Heteroglycan	Cell-cycle G0/G1 arrest	(62)
	*Phoma herbarum*	YCP	(1→4)-α-D-glucan	Immunomodulation: B cell	(63)
	*Trichoderma pseudokoningii*	EPS	Heteroglycan	Apoptosis induction	(64)

"→" means glycosidic bond and "*" means polysaccharides derivatives.

## 3 Anti-tumor activity

**Figure 1:**
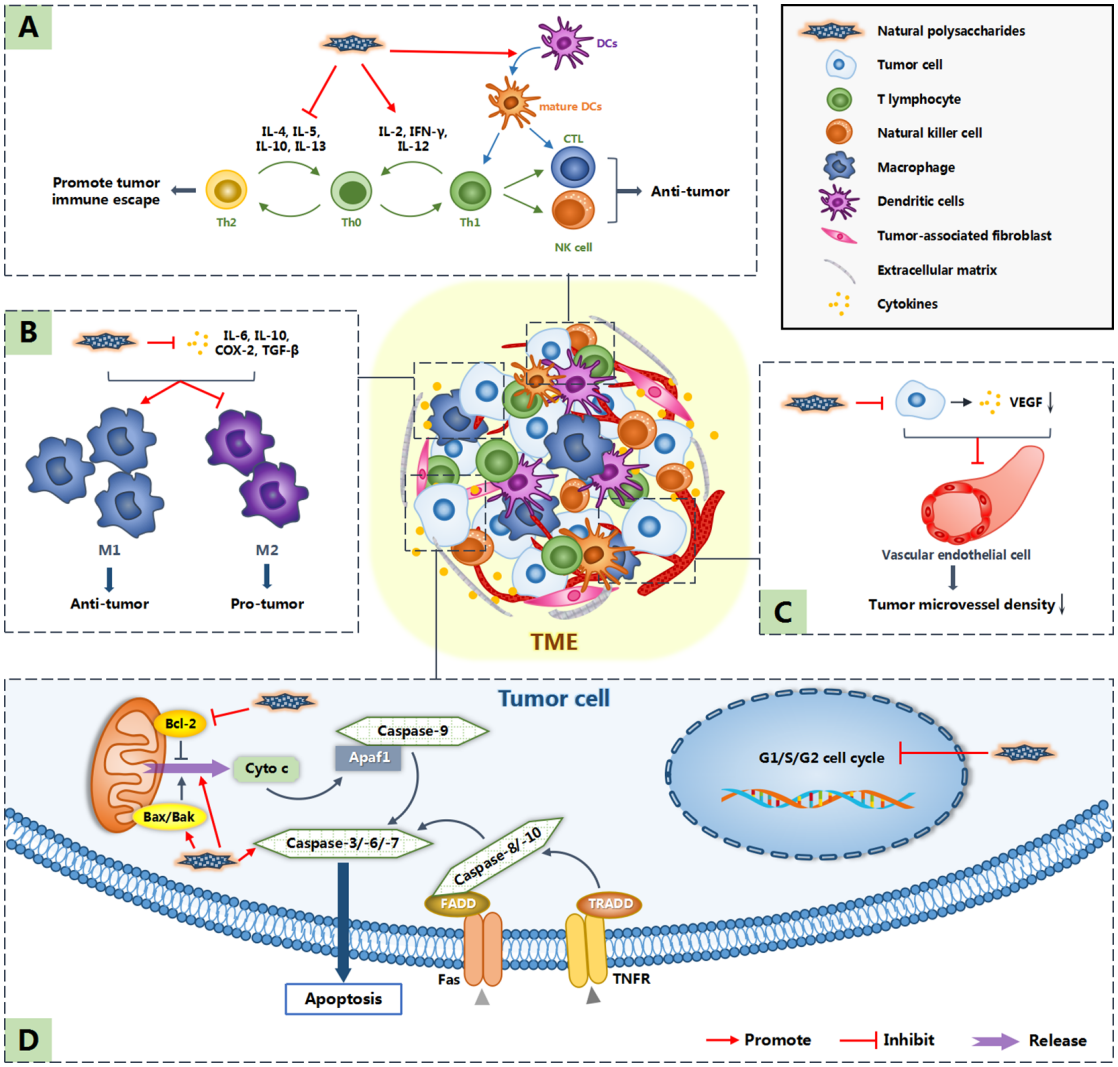
Anti-tumor mechanism of natural polysaccharides. Polysaccharides not only induce apoptosis by directly acting on tumor cells, but also inhibit the occurrence and development of tumors by acting on tumor microenvironment (TME). Among them, **(A)** indicates that natural polysaccharides promote the expression of cytokines such as IL-2, IFN-γ and IL-12, inhibit the expression of cytokines such as IL-4, IL-5, IL-10 and IL-13, resluting in promoting the differentiation of Th0 cells into Th1 cells, which have anti-tumor effect. What’s more, natural polysaccharides activate DC cells, allowing them to function normally in antigen presentation. **(B)** indicates that polysaccharide reduces the concentration of cytokines such as IL-6, IL-10, COX-2 and TGF-β in TME, so as to promote the differentiation of M1 macrophages,which play an anti-tumor role. **(C)** indicates that polysaccharides down regulate the expression of VEGF and so inhibit tumor related angiogenesis. **(D)** shows the direct anti-tumor effect of natural polysaccharides: cell cycle arrest and inducing apoptosis.

In addition to the original six characteristics, Professor Hanahan and Professor Weinberg added four features and summarized the ten hallmarks of cancer (66). They include sustaining proliferative signaling, evading growth suppressors, resisting cell death, enabling replicative immortality, inducing angiogenesis, activating invasion and metastasis, reprogramming energy metabolism, evading immune destruction, tumor-promoting inflammation and genome instability and mutation (66). These ten characteristics are also targets for tumor treatment. Natural polysaccharides are considered to inhibit tumor growth and metastasis by cell cycle arrest, inducing apoptosis, inhibiting angiogenesis and regulating host immune system (34, 36, 47, 56). In addition, the occurrence and development of tumors are not entirely attributed to the tumor cells themselves. In recent years, non-malignant cells and non-cellular components around tumor cells, namely tumor microenvironment (TME), have been increasingly proven to play an important role in the occurrence and development of tumors (67). Some polysaccharides can regulate the tumor microenvironment to indirectly realize their anti-tumor effect (59). **Figure 1** shows the possible anti-tumor mechanism of natural polysaccharides.

### 3.1 Cell cycle arrest

In normal cells, cell cycle arrest helps to maintain genome stability. By initiating cell cycle arrest, cells can avoid cell division in the process of stress and injury (68). Many natural polysaccharides are able to inhibit the proliferation of tumor cells by blocking cell division. This effect usually occurs in the interphase (G1, S and G2) of the cell cycle. For example, *Gayralia brasiliensis* polysaccharide Gb1 could cause cell cycle arrest in G1 phase, which prevents DNA replication from starting (47). And the over sulfurized product of Gb1, named Gb1-OS, can remarkably induce cell arrest in S phase and G2 phase (47). Another polysaccharide from *Salvia miltiorrhiza* (SMP) also has anti-proliferative effects against cancer cells by arresting cell cycle at S phase (52). p53 is a transcription factor, which is regarded to play a critical part in cell cycle arrest and apoptosis. The growth arrest of cells at the G1/S border is initiated by the destruction of cyclin D1 and Cdc25A, the activator of cyclin dependent kinase 2 (CDK2). p53 maintains this arrest by inducing the expression of CDK inhibitor p21 (69). Many tumor cells have been found to have mutations in p53 gene. Approximately 80% of p53 mutations are single point mutations with several hotspot mutations. In addition to losing function and dominant-negative effect on wild-type p53 activity, hotspot p53 mutant also obtained new oncogenic function (70). Wang et al. demonstrated that *Artemisia sphaerocephala* polysaccharide ASPs showed significant anti-tumor activity *via* inhibiting the expression of mutant p53 protein and inducing H22 Cell cycle arrest (42).

### 3.2 Apoptosis induction

Apoptosis is an autonomous and orderly cell death controlled by cellular signaling to maintain the stability of the internal environment. There are two main pathways of apoptosis: external or death receptor pathway and internal or mitochondrial pathway (71). The key process of apoptosis is the activation of caspases. Extrinsic pathway can be induced by members of the cytokine receptor TNF family, such as TNFR1 and Fas. These proteins recruit adaptor proteins to their cytosolic death domains (DDs) and then bind to death effector domain (DED)-containing pro-caspases, particularly pro-caspase-8 (72). The intrinsic pathway is induced by the release of cytochrome C from mitochondria. In the cytoplasm, cytochrome C binds and activates apoptotic protease activating factor-1 (Apaf-1) to bind and activate pro-caspase-9. The active caspase-9 and caspase-8 directly cleave and activate the effector protease, caspase-3, which finally start the apoptosis program (72). The control and regulation of mitochondrial pathway are almost related to Bcl-2 protein family. Among them, Bcl-2 plays an anti-apoptotic role by maintaining the integrity of mitochondrial membrane. In contrast, Bax and Bak can destroy mitochondrial membrane and promote the release of cytochrome C, thus activating caspase-9 (73).

Various natural polysaccharides have been found to induce tumor cell apoptosis by acting on mitochondrial pathway, as shown in **Figure 1D**. For instance, *Pleurotus ostreatus* polysaccharide Se-POP-3 (36), *Sargassum fusiforme* polysaccharide SFPS (5) and *Trichoderma pseudokoningii* polysaccharide EPS-1 (64) can increase the expression of Bax and reduce the expression of Bcl-2, so as to promote apoptosis. Additionally, Liu et al. reported that HSP-III, separated from *Hirsutella sinensis*, can collapse the mitochondrial membrane potential, release of cytochrome C, activate caspase-3 and caspase9, and finally induce the apoptosis of human non-small cell lung cancer H1299 cells (61). A proteoglucan from *Grifola frondosa* (PDF) has been proven to show strong anti-cancer activity in breast cancer cells through directly promoting the activation of caspase-7 and caspase-1, and increasing the expression of BAK-1 gene (74).

### 3.3 Anti-oxidation function

Oxidative stress is the result of the imbalance between the production of reactive oxygen species (ROS) and cell antioxidant defense, which is implicated in the etiology of cancer (75). In other words, chronic and cumulative oxidative stress will induce harmful modifications to various macromolecules, such as DNA (76). And DNA damage is considered as one of the mechanisms of tumorigenesis. Studies have manifested that antioxidants can help reduce cancer risk (77) and effectively prevent cancer. In addition to preventive effects, antioxidant supplementation during chemotherapy can reduce the toxic and side effects of chemotherapeutic drugs that may lead to ROS production (78). It must be noted that the concentration of the supplied antioxidants is crucial; a high concentration could cause an opposing effect (79). Careful control of the dose of antioxidants administered to treat some cancers could facilitate ROS scavenging, restore the redox balance in tumor cells, and abate their growth advantage (79). A few fungal polysaccharides, such as *Lentinus fusipes* polysaccharide (4) and *Tricholoma Lobayense* polysaccharide (40), have been found to eliminate superoxide anion and hydroxyl radical *in vivo*, prevent nucleic acid damage and inhibit the proliferation of tumor cells to some extent. Furthermore, He et al. studied the antioxidant activity of several animal-derived polysaccharides. The results indicated that both *Philomycus bilineatus* polysaccharide (58) and *Edwardsia sipunculoides* polysaccharide (6) had free radical scavenging activity in a dose-dependent manner.

### 3.4 Tumor microenvironment modulation

Tumor microenvironment (TME) provides essential support for tumor growth and development. The exact composition of TME varies among different types of tumors and different stages of tumors. Generally, TME consists of immune cells, stromal cells, blood vessels, and extracellular matrix (ECM) (80). Natural polysaccharides possess a wide range of immune activations, which makes them have broad application prospects in local tumor therapy targeting TME (81).

Macrophages are important immune cells in TME. They can be divided into inflammatory M1 macrophages with good antigen-presenting ability and cytotoxicity and immunosuppressive M2 macrophages involved in wound healing. TME promotes M2 phenotype through hypoxia and secretion of cytokines to support tumor growth and progression (80). Wang et al. found that *Antrodia camphorata* polysaccharide could reduce IL-6, IL-10, COX-2 and TGF-β in the TME of tumor bearing mice and then promote the transformation of tumor-associated macrophages (TAMs) to M1 type, so as to restrict tumor growth (82). The effect of natural polysaccharides on macrophages in TME is shown in **Figure 1B**.

Tumor infiltrating lymphocytes (TILs) are another major class of immune cells in TME. Polysaccharides usually achieve anti-tumor effects by regulating the ratio of Th1/Th2, as seen in **Figure 1A**. Th1 is a pro-inflammatory CD4^+^ T cell that activates and promotes the proliferation of CD8^+^ T cells and natural killer (NK) cells by secreting IL-2 and IFN-γ. Th2 mainly secretes IL-4, IL-5, IL-10 and IL-13, and first induces humoral immunity. More and more evidences show that Th2 cytokines play a significant role in mediating tumor immune escape, while Th1 cytokines are the main immunomodulatory cytokines with anti-tumor properties (59, 83, 84). For instance, *Ganoderma atrum* polysaccharide PSG-1 can increase the production of IL-2, IFN-γ and IL-12 (28), thereby promoting the differentiation of Th0 into Th1. A polysaccharide-protein complex from *Scolopendra subspinipes mutilans L. Koch* (SPPC) has a similar effect, which can markedly enhance the ratio of Th1/Th2 cytokines. At the same time, SPPC can also inhibit the expression of IL-10 and TGF-β, resulting in increasing the production of M1 macrophages (59).

Tumor cells affect or limit the function of dendritic cells (DCs) antigen presentation *via* releasing growth factors and cytokines, such as vascular endothelial growth factor (VEGF) and IL-10, which finally leads to tumor immune escape (12). Thus, even with sufficient numbers of DCs infiltrating the tumor microenvironment, they do not perform their normal functions. *Astragalus* polysaccharide (APS) has been found to promote DCs activation by increasing the expression of some immune-related suppressors such as CD86, CD80 and MHC-II on the DCs surface, resulting in enhanced interaction between DCs and T cells (85). The immunomodulatory function and mechanism of polysaccharides will be discussed with more details in the next section.

Furthermore, in order to overcome the hypoxic and acidic microenvironment, TME coordinated a procedure to promote angiogenesis to restore oxygen/nutrient supply and remove metabolic waste (80). A water-soluble polysaccharide from *Sargassum fusiforme* (SFPS) can reduce the expression of CD31, VEGF-A in SPCA-1 cells and so decrease tumor microvessel density (MVD) (54). Liu et al. also proved by immunohistochemical analysis that two sulfated derivatives of α-glucan from *Phellinus ribis* (PRP-S1 and PRP-S2) are able to inhibit tumor angiogenesis by down regulating the expression of VEGF in H-22 tumors (34). **Figure 1C** outlined the process of natural polysaccharides inhibiting tumor-related angiogenesis.

## 3 Immunomodulatory

**Figure 2:**
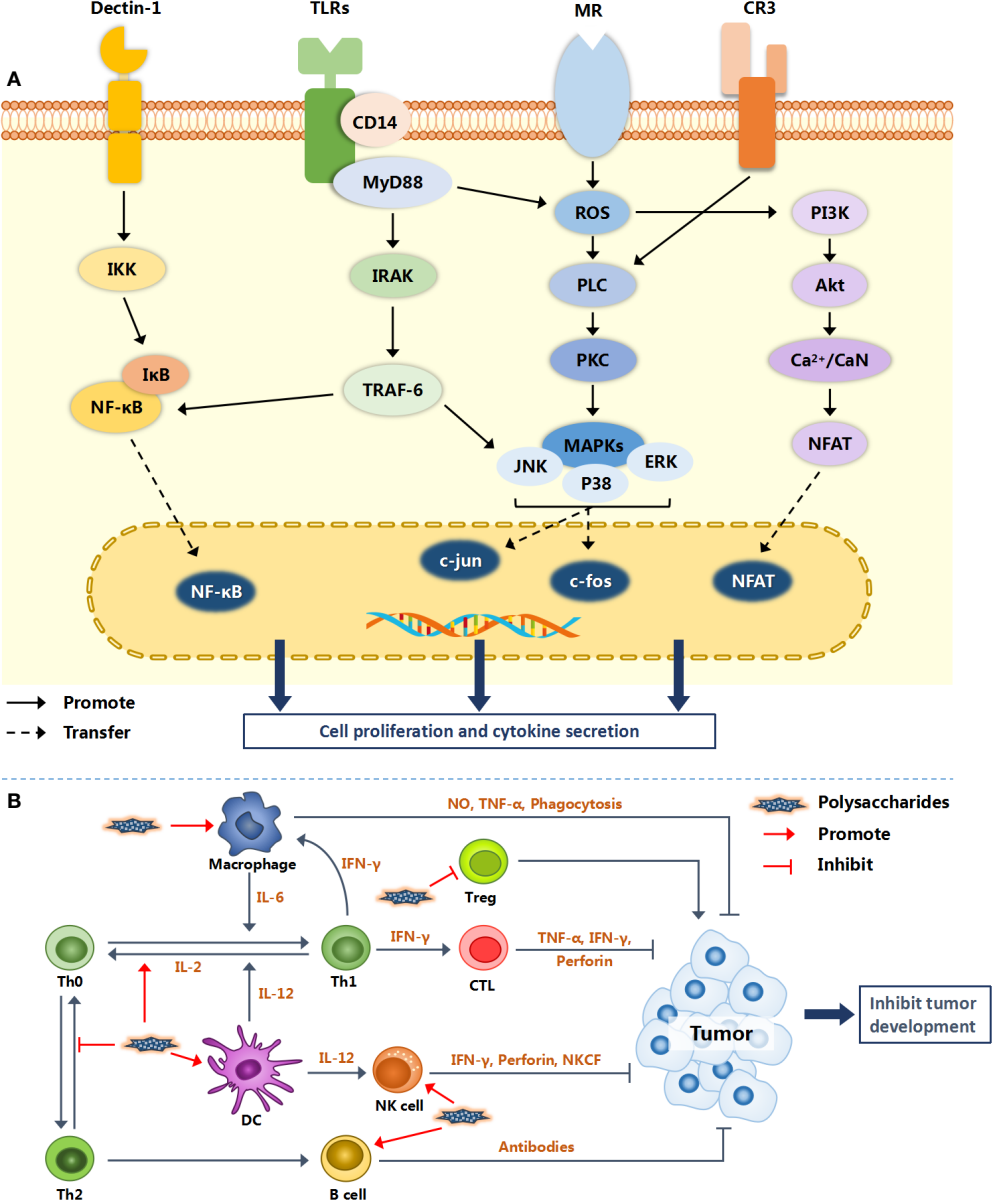
Immunomodulatory mechanism of natural polysaccharides. Natural polysaccharides activate intracellular signaling pathways *via* a variety of surface receptor binding modes (TLRs, Dectin-1, MR and CR3), and finally promote the proliferation and activation of immune cells [shown in **(A)**]. Various immune cells interact with each other to form an immune regulation network, resulting in inhibiting the growth and metastasis of tumor [shown in **(B)**].

More and more natural polysaccharides have been widely studied and applied being regarded as a class of immune-stimulant. They not only activate the immune response by combining mononuclear phagocytes (monocytes and macrophages) and antigen presenting cells (APCs), but also directly act on immune cells such as lymphocytes (T, B, NK cells) and neutrophils (86). It is noteworthy that the regulation of polysaccharides on immune cells is not carried out alone. Put differently, the same polysaccharide may activate different immune cells at the same time, therefore triggering a multi-channel anti-tumor mechanism. The specific action mechanisms of different polysaccharides are different. **Figure 2** illustrates the possible molecular mechanism of natural polysaccharides regulating the immune system.

### 4.1 Macrophages and DCs

The mechanism of polysaccharides on macrophages is relatively clear. In summary, they can promote the proliferation of macrophages, enhance the phagocytosis of macrophages, and stimulate the release of cytokines such as NO, TNF-α and IL-6 (87–89). Macrophages mainly bind to polysaccharides or their derivatives through toll like receptors (TLR2 and TLR4), CD14, mannose receptor (MR) and Dectin-1 receptor (10, 29, 86). The activation of macrophage receptors can initiate a series of intracellular signal cascades, leading to the transcriptional activation and production of inflammation related cytokines (**Figure 2A**).

Hsu’s team cloned the extracellular domains of 17 receptors into Fc fusion proteins, and then detected whether they interacted with polysaccharides by enzyme linked immunosorbent assay (ELISA). The results showed that the receptors which could interact with *Ganoderma lucidum* polysaccharide GLPs included Dectin-1, DC-SIGN, langreen, Kupffer cell receptor, macrophage mannose receptor, TLR2 and TLR4 (90). Dectin-1 is a C-type lectin-like receptor, which recognizes glucans linked by β-1,3 and/or β-1,6 glycosidic bonds (91) and mediates the biological effects of β-glucans as immune cell activators (92). The response of these cells to β-glucans requires the cytoplasmic tail and immunoreceptor tyrosine-based activation motif (ITAM)-like domain of Dectin-1, some of which involve collaboration with other signaling molecules, such as toll like receptor, TLR-2 and the myeloid differentiation factor 88 known as MyD88 (92). The polysaccharides isolated from *Cordyceps militaris* can induce up regulation of NO, ROS, TNF-α and phagocytosis by mitogen-activated protein kinases (MAPKs) and nuclear factor kappa-B (NF-κB) signaling pathways through Dectin-1 and TLR2 receptors in macrophages (93).

MR is another member of C-type lectin-like receptor family, which can recognize many sugar molecules containing mannose or focusing residues (94). The combination of polysaccharides and MR can increase the phagocytic activity of macrophages, produce ROS, activate transcription factor NF-κB and induce the secretion of cytokines (10).

Toll like receptors (TLRs) take a pivotal part in both innate immune system and acquired immune system, and are one of the hotspots involved in the function of immune regulatory receptors (94). After binding with TLR4 and other receptors, polysaccharides can activate PKC, MEK1, PAK and MAPKs, and transduce different signal cascades, leading to different biochemical reactions, such as the production of a variety of cytokines (95). TLR4 and TLR2 ligation brings about the activation of IL-1R associated kinase (IRAK) *via* an adaptor MyD88, with subsequent activation of TNF receptor-associated factor 6 (TRAF-6), MAPKs (p38 and JNK) and NF-κB. It can also activate phosphoinositide-3-kinase (PI3K)-Akt pathway *via* reactive oxygen species (ROS) signal, then promoting activation of MAPKs (10). Finally, these activators enter the nucleus and induce the expression of cytokines such as TNF-α, IL-6 and inducible nitric oxide synthase (iNOS). Lipopolysaccharide (LPS) is the cell wall component of Gram-negative bacteria. TLR4 has been considered as the only immune receptor of LPS for decades (96). LPS activates macrophages by binding to TLR4, and IL-1β, IL-10, IFN-γ and IL-6 are important markers to evaluate LPS-stimulated macrophages (56, 97). Apart from LPS, TLRs showed a wide range of affinity for a variety of natural polysaccharides. For example, both *Astragalus* polysaccharide APS (98, 99) and *Tinospora cordifolia* polysaccharide G1-4A (56) can stimulate macrophages by activating p38, ERK and JNK MAPKs in a TLR4-MyD88 dependent classical manner.

The surface distribution of receptors on DC cells is similar to that on macrophages, which provides prerequisite for polysaccharides to activate DCs. For instance, a polysaccharide from *Polyporus umbellatus* (PPS or PUP) can up regulate the co-expression of CD86 and CD11c on bone marrow DCs (BMDCs) *via* TLR4, and significantly induce BMDCs to produce IL-12, which is the most powerful stimulator for NK cells activation (37). In another experiment, after treatment with a polysaccharide from *Codium fragment* (CFPs), the concentrations of IL-6, IL-12 and TNF-α in BMDCs medium increased evidently, which promoted the activation of mouse DCs, NK cells and T cells (44). Additionally, the combination of CFPs sin immunotherapy can improve the anti-tumor effect of anti-PD-L1 antibody on lung cancer in animal model (100).

### 4.2 Lymphocytes

T cells originate in the bone marrow and mature in the thymus. In the thymus, T cells proliferate and differentiate into helper, regulatory, or cytotoxic T cells or develop into memory T cells. PSG-1, as mentioned above, is a homogeneous protein-bound polysaccharide and has been proven to enhance the proliferation of T lymphocytes. PSG-1 can not only elevate intracellular Ca^2+^ concentration and calcineurin (CaN) activity but also raise the p-ERK, p-JNK, and p-p38 expression levels (28). Yu et al. found that PSG-1 may induce the activation of spleen lymphocytes at least in part through Ca^2+^/CaN/NFAT/IL-2 signaling pathway and PKC/NFAT/IL-2 signaling pathway synergistically (30). Regulatory T cells (Tregs) have immunosuppressive effects, usually inhibiting or down regulating the induction and proliferation of effector T cells. Treg cells deficiency may lead to autoimmune diseases. However, the high frequency of Treg cells present sin tumor infiltrating lymphocytes (TIL) population often indicate poor clinical prognosis (101). *Codonopsis pilosula* polysaccharide CPPS could suppress excessive Tregs *via* surface receptor TLR4 mediated signaling pathway, and trigger a shift of Th2 to Th1 with activation of CD4^+^ T cells (46).

Both NK cells and neutrophils can interact with polysaccharides through complement receptor 3 (CR3) and Dectin-1 receptor. CR3 is a member of the β2 integrin family and consists of CD11b and CD18 domains. CR3 mediates many different important functions including leucocyte adhesion, activation, recruitment, host defense, phagocytosis and immune tolerance functions through interactions with numerous ligands such as iC3b, ICAM-1 and fibrinogen (102). A β-glucan from *Ganoderma lucidum* (GLP) can initiate innate immunity by binding CR3 on NK cells and directly activate neutrophils, eosinophils and T cells or B cells (103). Another experiments have proved that GLP can stimulate the production of cytolytic proteins (perforin and granule protein), up regulate the expression of NKG2D/NCR cell surface receptors, and activate intracellular MAPK signal (104). Moreover, Huang et al. developed a polysaccharides mixture consisting of GLP and PUP in a ratio of 3:1 (named GPP) and explored the biological activity of the mixture. The results showed that GPP significantly enhanced the function of RAW264.7 macrophage cells line and the activity of primary NK cells (105).

A few polysaccharides can directly stimulate the proliferation and activation of B cells. For example, *Alfalfa* Polysaccharide APS can effectively and selectively activate B cells and promote the production of IgM *in vitro*. This effect is mainly achieved through TLR4/MAPK/p38 pathway (41). In another experiment, a homogenous polysaccharide from the mycelium of marine fungus *Phoma herbarum* (YCP) was also foundto interact with TLR2 and TLR4 to activate p38, ERK and JNK in cells and transfer the transcription factor NF-κB into the nucleus, which finally led to the proliferation of B cells and the increase of IgM (63).

## 5 Clinical application

Natural polysaccharides increasingly show their clinical prospects in the field of anti-tumor and immune regulation for their easy extraction, low toxicity and changeable structures. More and more polysaccharides products have been used in combination with traditional chemotherapy drugs in order to enhance efficacy or reduce toxicity, which is also called immunochemotherapy (106). In Asia, due to the broad influence of traditional Chinese medicine, macrofungi have been collected, cultivated, eaten and used for medical purposes for at least 2,000 years. A variety of polysaccharides extracted from fungi have become routine clinical drugs. Among them, PSP and PSK, two commercial polysaccharides products from *Coriolus versicolo*r, are widely used in China and Japan, respectively.

PSP is a commonly used adjuvant drug for cancer chemotherapy or radiotherapy in China (107). It has been proven that PSP enhanced the cytotoxicity of etoposide (VP-16) on human breast cancer cells by interfering with S-phase progression and DNA synthesis (108). Another study by the same authors found that PSP could increase the sensitization of HL-60 cells to effective apoptotic cell death induced by Camptothecin, suggesting that PSP is a potential adjuvant in the treatment of human leukemia (109). Jin et al. combined PSP and *Astragalus* polysaccharide APS into a new complex prescription (PSP + APS), then they found that PSP+APS could restore the immunological effects against adriamycin (AMD) induced immunosuppression, such as the subset of leukomonocyte, the expression of IL-2/IL-2R in the spleen, and the thymus index (110).

As a non-specific immune stimulant, PSK has been used as an adjuvant therapy for gastric and colorectal cancer in Japan for many years (111). A systematic review and network meta-analysis showed that PSK combined with chemotherapy could significantly improve overall survival and disease-free survival without increasing side effects. The analysis suggested that PSK could be used as a first-line adjuvant immunochemotherapy drug in the clinical treatment of patients with gastroenteric cancer (106). Another systematic review indicated that when PSK was used as adjuvant treatment after standard chemotherapy, radiotherapy or surgery, it could prominently improve the immune function, tumor-related symptoms and survival of patients with lung cancer (112). In addition, Yamasaki et al. reported that PSK suppressed Hedgehog signaling through down-regulation of mastermind-like protein 3 (MAML3) and recombination signal binding protein for immunoglobulin-kappa-J region (RBPJ) transcription under hypoxia, inhibiting the induction of a malignant phenotype in pancreatic cancer (113), which provides a new idea for the treatment of refractory pancreatic cancer.

Similarly, polysaccharides from other sources are gradually accepted for clinic use. For example, dozens of polysaccharides products, such as *Poria cocos* polysaccharide, *Ganoderma lucidum* polysaccharide and *Grifola frondosa* polysaccharide, have been approved by China food and Drug Administration (SFDA) for chemotherapy or radiotherapy of a variety of cancers, hepatitis and other diseases (114–116).

More potential polysaccharides for immunochemotherapy are actively under research. Several polysaccharides have been shown to restore cyclophosphamide (CTX)-induced immunosuppression. CTX can reduce the activity of macrophages, promote macrophage apoptosis, and down regulate the levels of NO, IL-1β, IL-6 and TNF-α in macrophages. A natural high-methoxyl homogalacturonan from *Hippophae rhamnoide* (HRWP-A) was able to prolong the survival time of macrophages and inhibit their apoptosis. Meanwhile, HRWP-A significantly increased the levels of NO, IL-1β, IL-6 and TNF-α in peritoneal macrophages of CTX induced immunosuppressive mice (48). Polysaccharide from *Panax notoginseng* (NPPN) can not only directly inhibit the growth of H22 cells, but also improve the thymus index, cellular immunity, humoral immunity and bone marrow hematopoietic function of CTX induced immunosuppressive mice and bone marrow inhibitory mice (117). Hepatocellular carcinoma is closely related to hepatitis B virus. A novel polysaccharide from *Flammulina velutipes* (FVP1) has been proven to effectively inhibit the expression of HBeAg, HBsAg and HBV DNA replication in HepG2.2.15 cells, and has significant anti-HBV activity. This suggests that FVP1 may be used as a dietary supplement with immunomodulatory activity for HBV infection prevention (25).

## 6 Future prospects

The bioactivity of polysaccharides has been widely verified. On one hand, polysaccharides directly inhibit tumor growth and development through cell cycle arrest, apoptosis inducing, anti-angiogenesis and tumor microenvironment regulating. On the other hand, polysaccharides can also regulate the host’s immune system and indirectly play an anti-tumor role by stimulating non-specific immunity and specific immunity. Some molecular mechanisms of polysaccharides’ bioactivity have been clarified, but more in-depth research is needed to facilitate function-oriented polysaccharide drug screening and design. The structural variability of polysaccharides allows them to flexibly regulate some signaling pathways. However, it was also suggested that polysaccharides lack specific targets. As a result, based on current understanding, polysaccharides can only be used as broad-spectrum adjuvants rather than targeted drugs. More effort is required to be paid to the area of relationship between structures and function of polysaccharides in the future.

Additionally, the absorption and metabolism of polysaccharides are also important factors that affect their effective functioning. Polysaccharides usually exert pharmacological activities by oral administration. The absorption efficiency of polysaccharides after oral administration varies greatly and is mainly determined by factors such as charges (118), relative molecular mass (119), spatial structure (120) and dosage (121). Studies have found that the oral absorption of polysaccharides can be improved by structural modification of the polysaccharides (122) and the use of absorption enhancers (e.g. polyamines (123), chitosan (124) and thiolated polymers (125)). Exploring the best approach for improving the absorption would be beneficial for more effectively exerting the biological functions of polysaccharides.

## Author contributions

YY and WH wrote the paper. YY prepared the figures. All authors contributed to the article and approved the submitted version.

## Glossary

**Table:**

TME	tumor microenvironment
CDK2	cyclin dependent kinase 2
Cdc25A	activator of CDK2
TNF	tumor necrosis factor
TNFR1	TNF receptor 1
DDs	death domains
DED	death effector domain
Apaf-1	apoptotic protease activating factor-1
Bcl-2	B-cell lymphoma-2
Bax	Bcl-2 protein family member
Bak	Bcl-2 protein family member
ROS	reactive oxygen species
ECM	extracellular matrix
IL-n	interleukins-n
COX-2	cyclooxygenase-2
TGF-β	transforming growth factor-beta
TAMs	tumor-associated macrophages
TILs	tumor infiltrating lymphocytes
DCs	dendritic cells
BMDCs	bone marrow DCs
NK cells	natural killer cells
IFN-γ	interferon-gamma
VEGF	vascular endothelial growth factor
MHC	major histocompatibility complex
MVD	microvessel density
APCs	antigen presenting cells
TLRs	Toll-like receptors
MR	mannose receptor
ELISA	enzyme linked immunosorbent assay
ITAM	immunoreceptor tyrosine-based activation motif
MyD88	myeloid differentiation factor 88
MAPKs	mitogen-activated protein kinases
ERK/JNK/p38	subfamily of MAPKs
NF-κB	nuclear factor kappa-B
PKC	protein kinase C
MEK1	mitogen-activated extracellular signal-regulated kinase 1
IL-1R	IL-1 receptor
IRAK	IL-1R associated kinase
TRAF-6	TNF receptor-associated factor 6
iNOS	inducible nitric oxide synthase
LPS	lipopolysaccharide
PI3K	phosphoinositide-3-kinase
CaN	calcineurin
NFAT	nuclear factor of activated T cells
Tregs	regulatory T cells
CR3	complement receptor 3
ICAM-1	intercellular cell adhesion molecule-1
NKG2D	natural killer group 2D
NCR	natural cytotoxicity receptors
MAML3	mastermind-like protein 3
RBPJ	immunoglobulin-kappa-J region.
