# Corrigendum: Immunomodulatory function and anti-tumor mechanism of natural polysaccharides: a review

**DOI:** 10.3389/fimmu.2023.1361355

**Published:** 2024-01-09

**Authors:** Yang Ying, Wu Hao

**Affiliations:** ^1^ Cancer Institute, The Second Affiliated Hospital, Zhejiang University School of Medicine, Hangzhou, Zhejiang, China; ^2^ Key Laboratory of Cancer Prevention and Intervention, China National Ministry of Education, Key Laboratory of Molecular Biology in Medical Sciences, Hangzhou, Zhejiang, China; ^3^ Zhejiang Provincial Clinical Research Center for Cancer, Cancer Center of Zhejiang University, Hangzhou, Zhejiang, China

**Keywords:** polysaccharides, anti-tumor, tumor, immunomodulatory, immune response

In the published article, there was an error in the reference list. The reference list was incorrectly revised after the in-text citations were renumbered to conform with Frontiers journal style.

The correct reference list appears below.
